# Creating Robust Antimicrobial Materials with Sticky Tyrocidines

**DOI:** 10.3390/antibiotics11020174

**Published:** 2022-01-28

**Authors:** Wilma van Rensburg, Marina Rautenbach

**Affiliations:** BIOPEPTM Peptide Group, Department Biochemistry, Stellenbosch University, Stellenbosch 7602, South Africa

**Keywords:** antimicrobial peptides, tyrocidines, self-sterilising materials

## Abstract

Modified antimicrobial and antifouling materials and surfaces can be used to limit the propagation of microorganisms on various surfaces and minimise the occurrence of infection, transfer, and spoilage. Increased demand for ‘green’ solutions for material treatment has pushed the focus towards to naturally produced antimicrobials. Tyrocidines, cyclo-decapeptides naturally produced by a soil bacterium *Brevibacillus parabrevis*, have a broad spectrum of activity against Gram-positive and Gram-negative bacteria, filamentous fungi, and yeasts. Continual losses in tyrocidine production highlighted the possible association of peptides to surfaces. It was found in this study that tyrocidines readily associates with many materials, with a selectivity towards polysaccharide-type materials, such as cellulose. Peptide-treated cellulose was found to remain active after exposure to a broad pH range, various temperatures, salt solutions, water washes, and organic solvents, with the sterilising activity only affected by 1% SDS and 70% acetonitrile. Furthermore, a comparison to other antimicrobial peptides showed the association between tyrocidines and cellulose to be unique in terms of antimicrobial activity. The robust association between the tyrocidines and various materials holds great promise in applications focused on preventing surface contamination and creating self-sterilising materials.

## 1. Introduction

Surface contamination with microorganisms affects multiple industries throughout the production and supply chain. Persistent microbial contamination results in product losses, and inadequate treatments can promote resistance in plant and human pathogens with increased health risks, which, in totality, can put a financial strain on any industry. It is, therefore, crucial to limit surface microbial contamination as much as possible as this leads to secondary transfer and spread of microbial pathogens.

The contaminated ‘surface’, as well as types of preventative and decontamination measures, differs from industry to industry. As an example: the fruit industry faces a list of post-harvest problems, such as bacterial and fungal infections, spoilage, drying/shri-velling, and moisture loss. Fortunately, most of these concerns are addressed with the use of edible coating for fruits [1,2,3,4]. In addition to the protective coatings, fruits are packaged to prevent bruising and, to some extent, moisture loss [5,6]. The concern, however, is that a baseline of infection in these packaging materials would lead to the spoilage of the very fruit it is supposed to protect. A large number of fruits produced in the Southern hemisphere are exported to European markets [7] with an increasing demand for environmentally friendly or ‘green’ products [1,8,9]. Therefore, there is a need for an antimicrobial agent that would continually sterilise the packing material that is not a harsh and/or leaching chemical but falls within the scope of a ‘green biocide’. Recent environmental studies have also stressed the hazardous impact plastic consumables have regarding the manufacturing of plastic, as well as the negative effect of waste on wildlife, especially marine animals [10,11,12]. Possible solutions include biodegradable plastic-mimicking substitutes; however, due to their biodegradable nature, these materials are susceptible to microbial contamination that would affect their integrity. As previously mentioned, legislation is moving towards the implementation of ‘green solutions’; therefore, it is beneficial if a green biocide can prolong the use of these materials. Antimicrobial peptides are likely candidates to be used in the protection of food and the creation of novel antimicrobial material, surfaces, and self-sterilising packaging. Nisin is one of the few antimicrobial peptides with GRAS (Generally Recognised As Safe) approval for food preservation [5,13,14,15] and at the centre of antimicrobial surface development research, especially cellulose-based packaging and wound dressings [16,17,18,19,20,21,22]. The drawback of nisin is that it is primarily targeted against Gram-positive bacteria [23], with limited Gram-negative antibiotic activity in the presence of additives only [24,25,26] and no fungal activity [23]. Unfortunately, resistance against nisin has been increasing over the last decade [27], so pro-active development of alternative antimicrobials is crucial. Therefore, other antimicrobial peptides are being considered for similar applications [28].

The tyrocidines and tyrocidine analogues (Trcs) are antimicrobial cyclo-decapeptides produced intracellularly by the soil bacterium *Brevibacillus parabrevis* during the late logarithmic growth phase [29,30,31,32,33] as part of a peptide complex with linear gramicidins (gramicidin D), collectively known as tyrothricin. Trcs has a broad spectrum of activity against Gram-positive bacteria, such as *Listeria monocytogenes* [34,35], *Staphylococcus aureus* [36], *Streptococcus hemolyticus* [36], *Micrococcus luteus* [34], and a broad range of environmental Gram-positive bacteria [8]. Furthermore, these cyclo-decapeptides have activity against the human pathogenic yeast *Candida albicans* [37] and filamentous fungi, such as *Fusarium ssp.* [8,38], *Botrytis cinerea* [38], *Aspergillus fumigatus* [39], and a broad range of fungal plant pathogens [8,39]. Trcs are primarily active against Gram-positive bacteria; however, they do have some activity against Gram-negative bacteria [8,40,41]. Activity against a variety of viruses have been also reported [42]. These peptides are mainly membrane-active [37,38,43], but other cellular targets have been reported [44,45,46]. The combination of multiple modes of action and the speed of activity [34,43] makes the development of resistance less likely [42,46]. This is indeed the case with tyrothricin, for which no resistance has been reported since its introduction to clinical applications more than 70 years ago [47,48,49]. The peptides are unfortunately haemolytic and leukocytolytic [50,51], limiting them to topical clinical applications. It must be noted that, in the more than 70 years that it has been used as a topical cream and in throat lozenges, no resistance could be found nor induced against the peptides in tyrothricin [52].

Continual losses in peptide yield during purification observed by our research group has highlighted the possible association of the Trcs to filters, plastic consumables and various chromatography resins. Juhl et al. [53] found that the tyrocidines had specific interactions with glucose, sucrose, and cellotetraose. Masoudi et al. [54] observed that the formulation of the tyrocidines with soluble celluloses stabilised their anti-Candida activity. We realised that this previously unwanted adsorption to materials could be harnessed to create broad-spectrum antimicrobial surfaces. The peptides themselves are heat-stable, pH-stable, and resistant to general proteolytic degradation [44,55], providing the needed stability for the industrial application of peptide-containing solid surfaces. Their membranolytic mode of action is also perfectly suited for the creation of an antimicrobial solid surface or self-sterilising materials since they can still come into contact with their target(s) on the cell surface of the microorganism. As such, a membranolytic agent can elicit antimicrobial activity while being associated with a surface, which is a key point to consider with the development of antimicrobial surfaces [56].

In this study, we utilised general laboratory-acquired and commercial materials to test the retention of tyrocidines and their surface-associated antimicrobial activity against the Gram-positive food-pathogen, *L. monocytogenes*. The stability of the tyrocidines on cellulose was also determined through exposure to heat, pH ranges, salt solutions, acidic solutions, acetonitrile, multiple water washes, and the surfactant, sodium dodecyl sulphate. Finally, the uniqueness of tyrocidine association to cellulose was compared to the activity of cellulose treated with other known antimicrobial peptides against *L. monocytogenes* and *Escherichia coli*.

## 2. Results and Discussion

### 2.1. Activity of Trc-Mix Treated Material against L. monocytogenes

**Figure 1:**
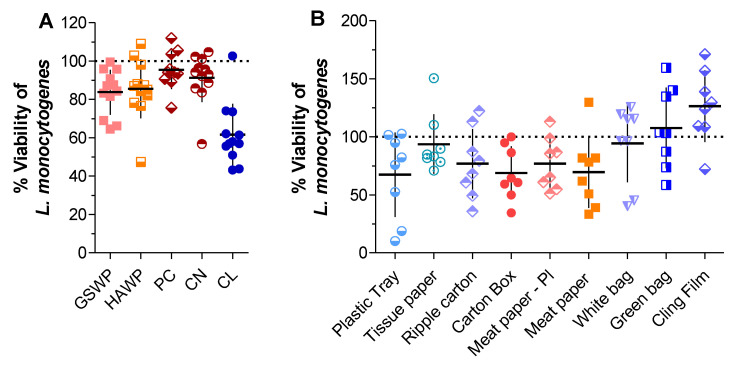
Effect of the base material on the metabolic activity of 5 × 10^6^ cells/cm^2^ *L. monocytogenes* expressed as percentage viability. (**A**) Laboratory acquired materials mixed cellulose filters GSWP (0.22 µm) and HAWP (0.45 µm); polycarbonate (PC—0.4 µm); cellulose nitrate (CN—0.45 µm) and cellulose (CL). (**B**) Commercially obtained cellulose-based and ‘plastic’ materials. Each data set represents the mean and SD (standard deviation) of eight and six technical repeats for the laboratory and commercial materials, respectively.

Development of antimicrobial materials first required the assessment of the base material (untreated or unmodified) in terms of the viability of the target organisms on these materials. We selected several materials to utilise as base materials as summarised in Table 1. The laboratory materials (Figure 1A) had some effect on the metabolic activity or viability of *L. monocytogenes* (expressed as percentage viability); however, the inhibition caused by untreated cellulose was more pronounced than that of the other materials. Similar results were observed for the commercially obtained materials (Figure 1B). The variability observed can be attributed to the materials moving within the well after PBS and resazurin were added, influencing the bacterial interaction and inhibition/stress and, therefore, the subsequent fluorescence detected. It is unclear why some materials are more antagonistic towards *L. monocytogenes,* especially since there is limited insight in the production of these materials. The antagonism can be attributed to various factors, including, but not limited to, residual chemical compounds from material production and the materials creating an environment that either halts multiplication or result in cell lysis.

The cell viability observed for the different plates (refer to Table 1; BP, PPR, PSR, AP) could not be compared since some of the plates are not typically suited for fluorescence, and the well design influences the overall fluorescence. The resazurin conversion of untreated wells with culture in each plate was, therefore, just used as a viability control and 100% survival to calculate the relative activity of the peptide associated to the plate. 

**Figure 2:**
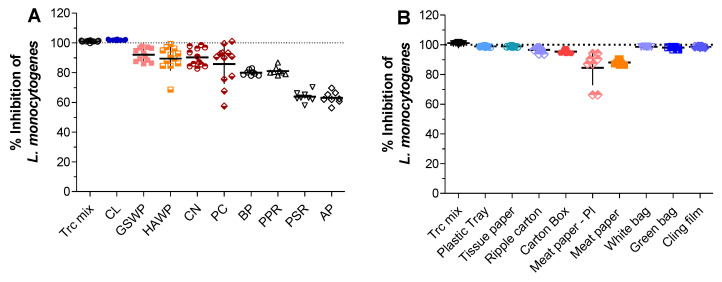
Antimicrobial activity against *L. monocytogenes* of Trc mix-treated (**A**) laboratory materials and (**B**) commercial materials used in the food industry. Each data set represents the mean of eight and six technical repeats for the laboratory and commercial materials, respectively. The error bar represents the SD of the data cluster. Abbreviations: cellulose (CL), mixed cellulose filters GSWP (0.22 µm) and HAWP (0.45 µm), cellulose nitrate (CN—0.45 µm), polycarbonate (PC—0.4 µm), black polystyrene plate (BP), polypropylene round bottom plate (PPR), polystyrene round bottom plate (PSR), and polystyrene plate (AP). Statistical analysis summary can be found in the Supplementary Materials Tables S2 and S3.

The antimicrobial activity of the Trc mix-treated materials (expressed as percentage inhibition) was calculated using the viability observed on its untreated counterpart as the viability control. Trc mix (5.0 µg dried in the plate) served as ‘full adsorption’ reference and the maximum activity that could be achieved for each of the treated materials. The Trc-treated cellulose fully inhibited the viability of *L. monocytogenes* (Figure 2A), followed by the Trc-treated GSWP, HAWP, CN, and PC, all having comparable activities.

Interestingly, the Trc-treated BP and PPR showed 80% inhibition and PSR and AP showed 60% inhibition. Though the activity observed increases the application of tyrocidines to a broader scope of materials, it does have some practical implications. The black plate (BP) is used for fluorescence studies of the peptide association to cellulose. PSR and AP are used in dose-response assays to determine Trcs’ activity against multiple target organisms [34,37,39]. The peptides’ association to the plates (based on the observed activity) would, therefore, have an influence on the observed activity within the assay. 

Full inhibition was observed for the Trc mix-treated plastic tray, tissue paper, ripple carton, white bag, green bag, and cling film (Figure 2B). The carton box showed high inhibition, followed by both sides of the meat paper, showing similar results. 

### 2.2. Determining the Amount of Trcs in Antimicrobial Materials

**Figure 3:**
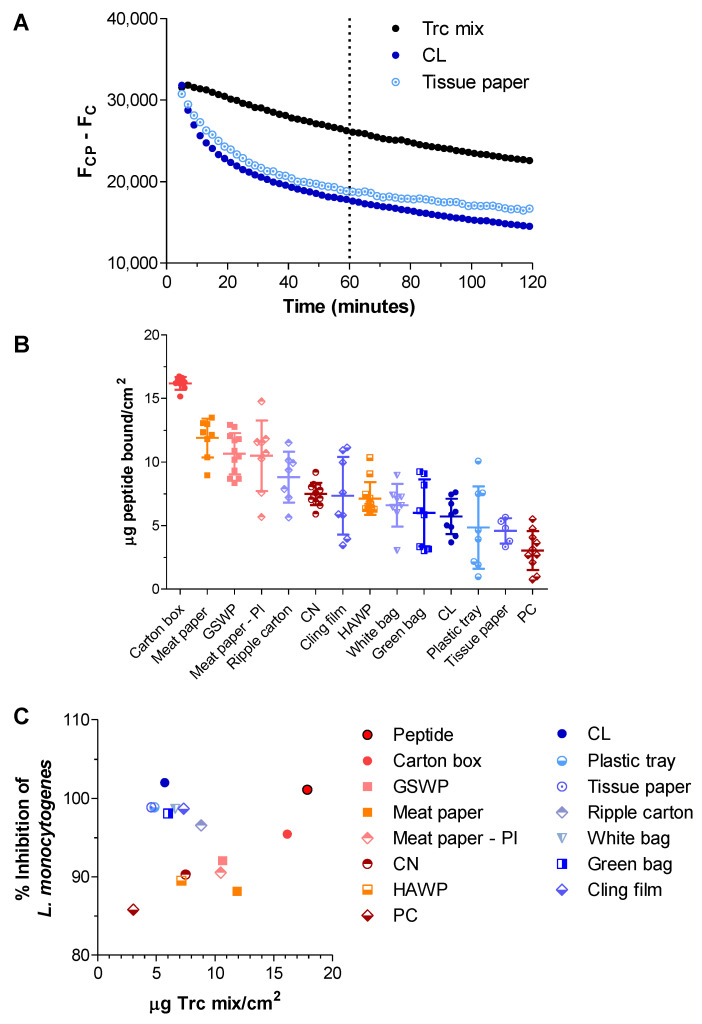
(**A**) Fluorescence decrease over time (background subtracted) as observed for Trc mix (50 µg/mL), with cellulose and tissue paper as examples. Time point show at 60 min as ‘end point’ based on fluorescence decrease. (**B**) Amount of peptide bound (µg/cm^2^) to each of the materials as calculated after 60 min of association. Each data set represents the mean of at least 6 technical repeats. The error bar represents the SD of the data cluster. Refer to Supplementary Materials for detail on concentration determination. (**C**) Relationship between peptide bound (µg/cm^2^) and activity (percentage inhibition of *L. monocytogenes*).

The amount of peptide associated with the selected materials was determined with the use of a fluorescence method tracking the movement of the fluorophores (Trp and Tyr) that occur in the tyrocidine structure. The full development and optimisation of the method is described in Supplementary Materials. The fluorescence signal studied was background subtracted in order to remove any influence on the fluorescence by the materials. It was observed that the fluorescence signal of Trc mix (50 µg/mL) alone decreases over time (Figure 3A), which can be caused by two factors: self-assembly/aggregation and the peptide binding to the plate well surface. Tyrocidines are prone to aggregation, especially in polar solvents [57,58,59], which would lead to aromatic stacking resulting in fluorescence quenching over time.

The second cause of the fluorescence decrease is the removal of the peptide from the solution due to the peptides’ association to a surface, in this case, the plate. This can be confirmed by the antimicrobial activity observed for the BP (Figure 2A). The addition of a material (e.g., cellulose or tissue paper) showed a much faster decrease in fluorescence which plateaued before or around 60 min of incubation (Figure 3A). This time point was, therefore, selected to estimate the amount of peptide bound to the surfaces. Consequently, the standard curve used to determine the limits of peptide detection, was determined at the same time point of 60-min incubation in the BP plate. 

Similar results were found for the other materials in this study. The carton box (Figure 3B) bound the maximum amount of peptide available that could be quantified based on the sensitivity of the fluorescence method. However, this treated material could not fully inhibit the viability of *L. monocytogenes*, probably because it consisted of multiple layers decreasing the true µg/cm^2^ coverage of Trc mix below the 3.5 µg/cm^2^ needed for full inhibition. The minimum amount needed to result in full inhibition is calculated based on the MIC of 1 µg peptide per 0.28 cm^2^ of sample size (results not shown). In the event of industrial application, this could possibly be rectified by increasing the peptide concentration at the incubation step or including the tyrocidines in the manufacturing process. 

The remaining results are summarised from the most peptide associated to the least (Figure 3B). A comparison between the activity of peptide-treated material (expressed as percentage inhibition) and peptide bound (µg/cm^2^) showed two distinct groups (Figure 3C). The first group has a seemingly linear relationship between peptide bound and activity (red/orange). The second group appears to cluster around 4–8 µg/cm^2^ resulting in 95–100% inhibition of *L. monocytogenes* (blue/green). Each of the base materials affected the viability of *L. monocytogenes* differently; therefore, no true comparison can be made regarding peptide association to various surfaces other than that tyrocidines are able to associate and remain active. There was also no clear selection for a type of material (e.g., cellulose materials having the best association and activity) as the two groupings contained both cellulose-based materials and plastics.

### 2.3. How Robust Is the Activity of the Trc-Cellulose?

Stability is one of the greatest concerns of using a biocide that had been isolated from nature without any further chemical modification in the industrial creation of antimicrobial solid surfaces since these processes can make use of harsh conditions. Just in the paper and packaging industries alone, heating steps are used to dry materials before storage; thus, any antimicrobial additive used should be stable at high temperatures. The tyrocidines and tyrocidine analogues are heat-stable when exposed to 90 °C for 10 min [44] and previous studies have shown that tyrocidine-treated cellulose remains active against *M. luteus* after exposure to up to 100 °C wet temperature for a minute [60,61]. This experiment was repeated against *L. monocytogenes* and extended to ‘dry’ heat exposure. Cellulose was chosen from the available base-materials as the peptide-treated cellulose had the highest activity of the laboratory acquired materials (Figure 2A), and as a representative material of the cellulose based commercial materials, as in the case with the food packing materials. The pre-treated cellulose was exposed to 100 °C, 125 °C, 150 °C, 175 °C, and 200 °C, for 1 and 10 min. Three peptide mixtures were tested: Trc mix (containing all the main tyrocidine analogues), a Phe supplemented culture extract (containing predominantly tyrocidine A (TrcA) and Phe-rich analogues, such as phenycidine A (PhcA)), and a Trp supplemented culture extract (containing predominantly tryptocidine C (TpcC) and Trp-rich analogues, such as tyrocidine C (TrcC)). 

**Figure 4:**
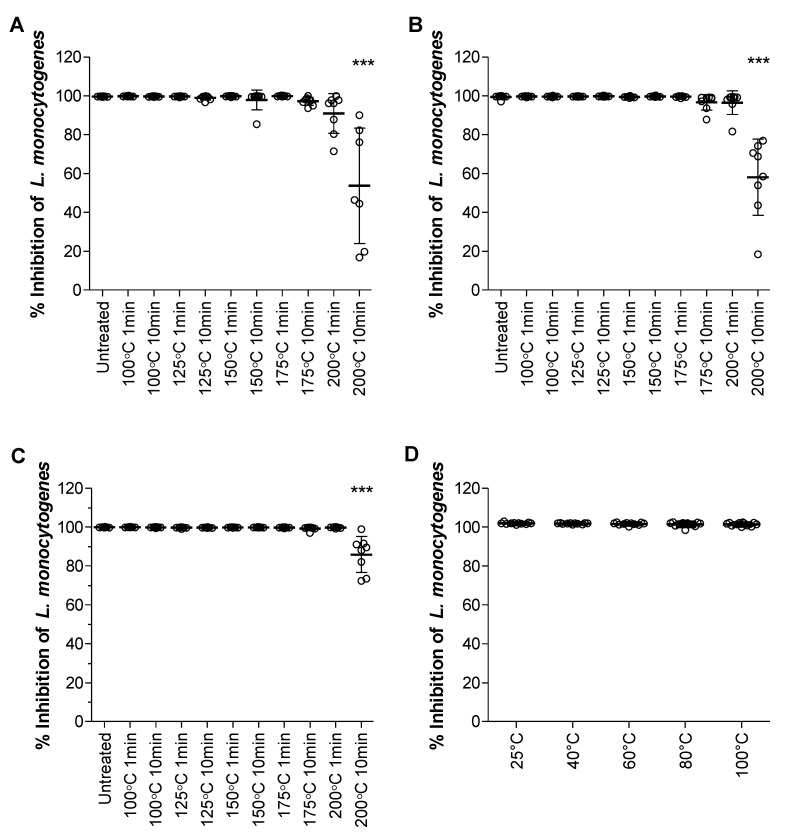
Antimicrobial activity of peptide-treated cellulose against *L. monocytogenes* (5 × 10^6^ cells/cm^2^) after exposure to ‘dry’ temperature ranges at 1 and 10 min Three peptide analogue mixtures were tested: (**A**) Trp supplemented culture extract, (**B**) Phe supplemented culture extract, and (**C**) Trc mix. (**D**) Antimicrobial activity of Trc mix-treated cellulose after exposure to ‘wet’ temperature ranges. Data represents the mean and SD of 9 repeats. Statistical analysis between untreated as control and the other conditions were determined with One-way ANOVA and Bonferroni’s Multiple comparison post-test; *** *p* < 0.001.

The Trc mix-treated cellulose maintained activity up until 10-min exposure to 175 °C (Figure 4C), which was also observed for the Phe-rich peptide-treated cellulose (Figure 4B). The same loss of activity at the 10-min exposure at 200 °C was observed for the Trp-rich peptide-treated cellulose (Figure 4A), and, even though the effect was not significant, there appears to be some activity loss for the cellulose exposed to a minute at 200 °C. Comparing the 10-min exposure at 200 °C of each of the peptide extracts, it appears, though there is no statistical significance, that the Trc mix-treated cellulose is less affected by the high temperatures than the cellulose treated with Phe-rich peptide complexes and Trp-rich peptide complexes (refer to Supplementary Materials, Figure S1 and Table S1). The first aspect to consider is that Trp is broken down at high temperatures [62,63,64,65] which, given the high amount of Trp containing analogues in the Tpcs-extract, would explain the decrease in activity at higher temperatures (Figure S1 and Table S1). However, this does not hold true for the commercial Trc mix and Phe-rich peptide complexes in which 27% and 59% of the respective extracts are Phe-containing peptide (TrcA, TrcA_1_, PhcA). This would relate to cellulose treated with Phe-rich peptide complex to be more temperature-stable, which it is not. The other factor to consider is the abundance of Tyr^7^ peptides (tyrocidines) within each extract: Trc mix contains 91% tyrocidines, whereas Phe-rich peptide extract contains various Phe containing peptides (Table S1).

It would, therefore, appear that heat stability can be attributed to both the presence of Tyr^7^ in the tyrocidines, and the identity of the amino acids present in the dipeptide unit. The activity of the ‘wet’ temperature exposed cellulose remained unaffected in comparison to the control (25 °C) against *L. monocytogenes* (Figure 4D). This correlates with what has been previously reported [60,61]. It can, therefore, be concluded that the peptide remains heat-stable, even though it is associated with cellulose. Furthermore, the peptide would maintain activity with high-temperature manufacturing and drying steps when incorporated in the production of antimicrobial cellulose-based materials.

**Figure 5:**
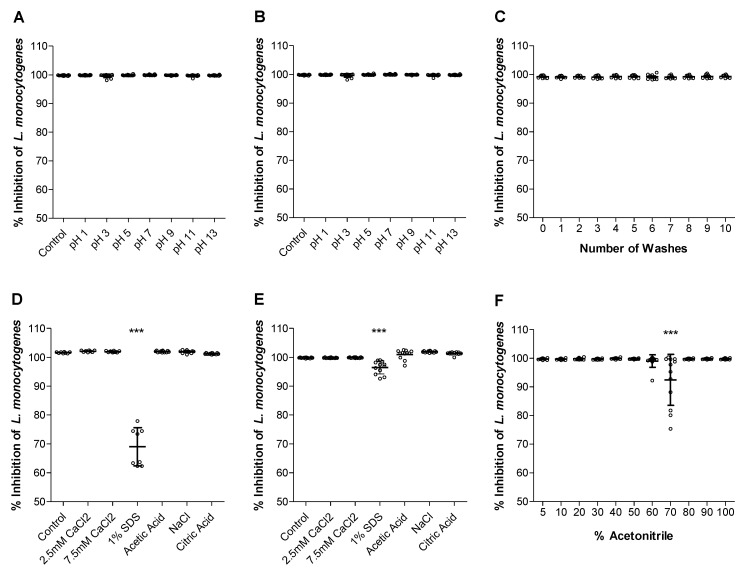
Antimicrobial activity, expressed in percentage metabolism inhibition against *L. monocytogenes*, of Trc mix (**A**,**D**) adsorbed and (**B**,**E**) desorbed in the presence of pH ranges, salts, 1% SDS, 5% *m*/*v* acetic acid, 5 mM NaCl, and 3% *m*/*v* citric acid. Antimicrobial activity of Trcs-treated cellulose exposed to (**C**) multiple water washes and (**F**) treatment with ranges of percentage (*v*/*v*) acetonitrile. Data represents the mean and SD of three biological and four technical repeats. Statistical analysis between untreated as control and the other conditions were determined with One-way ANOVA and Bonferroni’s Multiple comparison post-test; *** *p* < 0.001.

Determining solvent stability is dual purpose as it provides the conditions under which the Trcs-cellulose would remain active and offer some insight into the conditions that mediate the peptide-cellulose association. A study into the association between gramicidin S and spores showed that incubation with SDS fully inhibited association, leading to the conclusion that a combination of hydrophobic and electrostatic interactions determines association [66]. However, the addition of just Ca^2+^ (disrupts electrostatic interactions), ethanol (disrupts hydrophobic interactions), and liquids with pH higher than 10 (disrupts ionic and polar interactions) caused some dissociation but did not fully remove the active peptides [66]. Tyrocidines share 50% sequence similarity, namely the Val-Orn-Leu-D-Phe-Pro moiety, with gramicidin S and likewise play a pivotal role in spore germination [32,67]. CaCl_2_ has been shown to increase the activity of Trcs against *L. monocytogenes* [68,69] but reduced the activity against certain fungi [70]. It has also been shown to change the mode of action of the Trcs from lytic to non-lytic [69]. It was observed that pH did not affect adsorption (Figure 5A) nor cause desorption (Figure 5B) of Trcs in terms of a change in activity, and neither did CaCl_2_, acetic acid, NaCl, or citric acid (Figure 5D,E). 

The only observed changes were for 1% SDS, affecting adsorption more than desorption. After ten water washes, enough Trc mix still remained associated to the cellulose leading to full inhibition of *L. monocytogenes* metabolism (Figure 5C). Exposure to a range of acetonitrile concentrations showed that the activity of Trc mix-treated cellulose was affected by 60–70% (*v*/*v*) acetonitrile (Figure 5F). 

It appears that the amphipathic nature of the tyrocidine dimers [58,71] and oligomeri-sation driven by hydrophobic interactions could play a key role in association to cellulose since only SDS and 70% *v*/*v* acetonitrile could affect adsorption/desorption enough to affect the activity against *L. monocytogenes.* The solubility of the tyrocidines is the best, in 60–70% acetonitrile range; therefore, peptide is removed from the matrix. At the low and high organic solvent concentrations, the peptide and its amphipathic oligomers are less soluble and most probably form solvent-stable nanostructures and films on the cellulose surface. The formation of nanostructures by the tyrocidines and analogues have been shown by several investigators [57,58,59,72,73,74,75,76]. The effect of a change in solvent environment on peptide oligomerisation, and possibly on the association of peptide to cellulose, therefore, is specific to the tyrocidines. These exposure studies exemplified the robustness of the Trc-celluloses created in this study. This robustness in maintaining activity on particularly cellulose can also point to the type of interaction, namely interaction in oligomers and with the matrix allowing the release of peptide only in the presence of a target or amphipathic solution. 

### 2.4. Role of Type of the Active Compound on Surface Activity—Are Trcs Unique Sticky Peptides?

Antimicrobial peptides are favoured for food protection [28] and in the creation of antimicrobial surfaces with the increased demand for ‘green’ products [1,8,9]. Considering the ability of tyrocidines to associate with cellulose, it was determined whether the same effect would be observed for other antimicrobial peptides if treated the same way. We compiled a representative library of compounds (Table 2) and treated cellulose filters in the same way as described for the Trcs.

**Figure 6:**
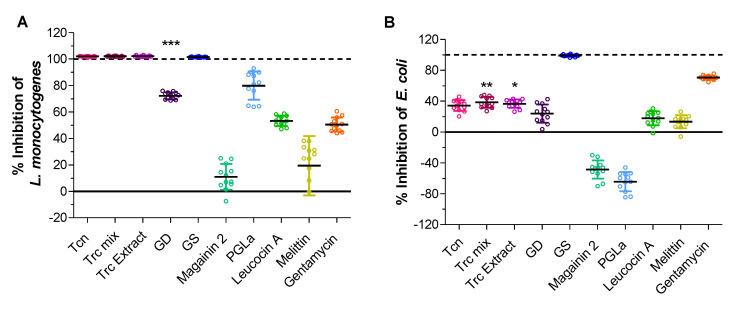
Antimicrobial activity (expressed as % Inhibition) of peptide-treated cellulose against (**A**) *L. monocytogenes* and (**B**) *E. coli*. % Inhibition is calculated/normalised by the natural activity of the base material cellulose. Gentamycin was used as antibiotic control. Each data set represents three biological and four technical repeats. Statistical analysis was performed with One-way ANOVA (Boferroni’s multiple comparison test) against GD versus Tcn, Trc mix, Trc extract, and for (**A**) *** *p* < 0.0001 and (**B**) ** *p* < 0.01 and * *p* < 0.05. Full statistical analysis can be found in the Supplementary Materials Tables S4 and S5.

We compared the purified Trc mix used in this study with commercial tyrothricin (Tcn) from which it was purified and a purified Trc peptide extract from an in-house bacterial culture extract. The first observation was that there is no difference in activity against *L. monocytogenes* between cellulose treated with Tcn, Trc mix, and Trc extract (Figure 6A). This holds great promise for the industrial application of Trcs-treated materials, since all the observations made of Trc mix-treated materials should be transferrable to cheaper Tcn and Trc extract-treated materials. Moreover, the linear gramicidins present in Tcn appeared not to have a major influence on the association of Trcs to the cellulose, or noticeably inhibit the activity of the treated celluloses. Linear gramicidins (GD) alone resulted in 70% inhibition of *L. monocytogenes*, and gramicidin S (GS) resulted in 100% inhibition. The four remaining peptides can be ranked, in terms of activity from most to least, as PGLa, leucocin A, melittin, and magainin 2. The activity for the control antibiotic, gentamycin, was comparable to that of leucocin A. Although it appears that some of the peptides are associated with cellulose, the resulting activity at the low concentrations used for Trcs, it is not comparable to the activity observed for Trcs, except for the related peptide GS. Furthermore, the stability or robustness of the association and activity will have to be confirmed before any more detailed comparison can be made. Previous studies [60] and results from this study showed that the antimicrobial activity of Trc-treated cellulose is highly robust and wash-stable to washes at up to 100 °C with water, temperatures up to 1 min at 200 °C, and up to 12 washes with water, as well as washes with solutions varying in organic solvent composition, pH, and salt content. This stability could relate to the specific association with cellulose [53] and the self-assembly character of the tyrocidines [57,58,59,71,74,77], leading to layering of the tyrocidines on the cellulose surface.

There was some activity of Tcn, Trc mix, Trc complex, and GD observed against *E. coli* (Figure 6B) which correlates to previous reports of these peptides’ specificity towards Gram-negative bacteria [40,41]. Interestingly, gramicidin S (GS) was the only compound that resulted in full inhibition, followed by gentamycin. Leucocin A and melittin resulted in low levels of inhibition, whereas PGLa and magainin 2 resulted in a stress response that is recorded as “negative” inhibition. A stress response can increase cell metabolism because of stress, such as osmotic stress, that is induced by the antimicrobial peptides [78]. 

Overall, it can be concluded that Trcs-containing cellulose is unique in terms of high activity against *L. monocytogenes* at low levels of peptide associated with the cellulose. Though only low levels of activity were observed for the Trcs-containing cellulose against *E. coli*, combinations with GS can be formulated to create a material that has a broad spectrum of activity against Gram-positive and Gram-negative bacteria. There is currently no research on GS-containing surfaces, however, cellulose is used as part of a purification method for GS [79,80,81], and the use of GS producing biofilms to prevent pipe corrosion has been reported [82]. The only other research on the activity of GS on materials was completed by our group, which showed GS having a comparable activity to Trc mix on cellulose against 10^5^ cells/cm^2^ *Micrococcus luteus* [60].

## 3. Materials and Methods

### 3.1. Materials

Gramicidin S, gramicidin D, tyrothricin, gentamicin, resazurin sodium salt, and KCl was supplied by Sigma (St. Louis, MO, USA). Acetonitrile, HPLC-grade far UV cut-off was supplied by Romil Ltd. (Cambridge, UK). Merck (Darmstadt, Germany) supplied agar, yeast extract, tryptone, Na_2_HPO_4_, and KH_2_PO_4_; and Merck (Wadeville, South Africa) supplied sodium chloride, brain heart infusion broth, and black 96-well polystyrene plates. From Corning (Kennebunk, ME, USA), 96-well polystyrene plates were acquired, and cellulose filters (Paper) (MN 615/No 1) were obtained from Macherey-Nagel (Düren, Germany). Mixed cellulose ester filters (HAWP, GSWP) were supplied by Waters-Millipore (Milford, MA, USA). Polycarbonate filters were supplied by Nuclepore Corp (Plesanton, CA, USA), and cellulose nitrate was provided by Sartorius (Gottingen, Germany). AcroPrepTM Advance 96 filter plates were sourced from Pall Corporation (Ann Arbor, MI, USA). Analytical grade water was obtained filtering water from a reverse osmosis plant through Millipore Milli Q^®^ water purification system (Milford, MA, USA). Melittin, magainin 2, leucocin A, and PGLa was sourced from of the BIOPEP^TM^ peptide library (Stellenbosch University, South Africa).

### 3.2. Selecting Base Materials

A range of laboratory and commercial materials was selected to study the association and resulting activity of tyrocidines (refer to Table 1). The laboratory materials included filters commonly used in solvent filtration: mixed cellulose GSWP (0.22 µm) and HAWP (0.45 µm), cellulose nitrate (CN) (0.45 µm), polycarbonate (PC) (0.4 µm), and cellulose (CL). In addition, included were plates used for peptide preparation and dose-response assays: black polystyrene plate used for fluorescence (BP), clear polystyrene plate (AP), clear round-bottom polypropylene plate (PPR), and clear round-bottom polystyrene plate (PSR). The commercial materials included fruit packaging materials: tissue paper, ripple carton, carton box, and a plastic tray. Biodegradable materials included: meat packaging paper that has a polylactate layer that is normally in contact with the meat (meat paper and meat paper PL), biodegradable polylactate plastics, namely a waste bag marketed for home use (white bag), an industrial grade waste bag (green bag), and cling film that is marketed for both home and catering purposes. 

### 3.3. Creation of Antimicrobial Materials 

An aqueous solution of antimicrobial compound (100 µL of 50.0 µg/mL; 5% *v*/*v* acetonitrile) was incubated for two hours with the base material punch (0.28 cm^2^), ensuring that 5.0 µg of compound was available for binding. Following the incubation, the antimicrobial compound solution was removed, and the samples washed twice with 100 µL sterile analytical quality water and dried in a 40 °C oven for 2 h. Treatment conditions were chosen to imitate large scale treatment/creation of packaging materials, such as aqueous solutions, followed by a drying step. The antimicrobial compounds used in this study are given in Table 2. 

### 3.4. Antimicrobial Activity of Trc-Containing Materials

The antimicrobial activity of the Trc-containing materials and films were determined by a developed resazurin assay [94]. *Listeria monocytogenes* B73 was streaked out from freezer stocks onto BHI agar plates (Brain Heart Infusion; 1.5% *m*/*v* agar) and incubated for 48 h at 37 °C until colonies were viable. *Escherichia coli* K12 was streaked out from freezer stocks onto LB plates (1% *m*/*v* NaCl, 1% *m*/*v* tryptone, 0.5% *m*/*v* yeast extract, 1.5% *m*/*v* agar in water) and incubated at 37 °C for 24 h. Starter cultures were prepared, by inoculating 1 mL medium with 3–5 colonies and growing the cells overnight at 37 °C at an angle, shaking at 150 RPM. The starter culture was sub-cultured into fresh media, grown until mid-exponential growth phase was reached at an OD600 = 0.4, which is 1.3 × 10^8^ cell/mL for *L. monocytogenes* and 4 × 10^7^ cells/mL for *E. coli*. This culture was then pipetted (10 µL) onto the treated and untreated materials in a 96-well assay plate and incubated for one hour at 37 °C. Following the incubation, 90 µL PBS (phosphate buffered saline; 0.8% *m*/*v* NaCl, 0.04% *m*/*v* KCl, 0.144% *m*/*v* Na_2_HPO_4_, 0.02% *m*/*v* KH_2_PO_4_; pH 7.4), and 10 µL resazurin dye (0.3 mg/mL in PBS) was added, followed by a further incubation at 37 °C. The conversion of the resazurin was measured at Ex_530_ and Em_590_ after 30 min for *L. monocytogenes* and 90 min for *E. coli* using the Tecan Spark 10 M Multimode Microplate Reader and controlled by the Spark Control^TM^ software, both provided by Tecan Group Ltd. (Mennedorf, Switzerland). Materials remained in the well as it did not interfere with fluorescence measurements. The activity of the material was determined in terms of percentage inhibition by using the acquired fluorescence readings (F), with the following Equation:

(1) $$\%Inhibition\;of\;Target\;organism=100-\frac{\left(F\;of\;well-F\;of\;average\;blank\right)\times 100}{\left(F\;of\;growth\;control-F\;of\;average\;blank\right)}.$$

The effect of the base materials on cell viability was determined by calculating the %*viability* from the resazurin conversion in the BP as viability control, compared to the resazurin conversion on the untreated base materials, with the following equation:

(2) $$\%Viability\;of\;Target\;organism=\frac{\left(F\;of\;well-F\;of\;average\;blank\right)\times 100}{\left(F\;of\;growth\;control-F\;of\;average\;blank\right)}.$$

As the characteristics of the assay plates (refer to Table 1; BP, PSR, PPR, AP) hinders fluorescence, direct comparisons of viability could not be made. The %*viability* on these plates only served to determine as the 100% relative survival to determine the relative activity of the treated wells in the plates after exposure to Trcs.

### 3.5. Determining the Amount of Trcs in Antimicrobial Materials

A fluorescence method was developed tracking the naturally occurring fluorophores found in the tyrocidine structure (Trp at position 3 and/or 4 and Tyr at position 7). Optimisation of the method and standard curve used to calculate the amount of peptide bound can be found in the Supplementary Materials. All fluorescence results were background subtracted to remove any effect the materials had on the fluorescence signal. 

The incubation step used for the creation of the antimicrobial materials, was assessed to determine the rate of association of the peptides to the base material. Fluorescence readings were collected at Ex_290_, and Em_342_ for two hours by using the Tecan Spark 10 M Multimode Microplate Reader which is controlled by the Spark Control^TM^ software, both provided by Tecan Group Ltd. (Mennedorf, Switzerland).

### 3.6. Heat Exposure of Trc-Cellulose

The effect of ‘dry’ temperature exposure on the activity of Trcs was done by treating cellulose disks with three different mixtures of peptide: Trc mix (containing a mixture of the primary tyrocidine analogues), a Phe-extract (containing Phe-rich peptides, such as Trc A/A1, phenycidine A/A1, and tryptocidine A/A1), and a Trp-extract (containing Trp-rich peptides, such as the Trc C/C1, Trc B/B1, and tryptocidine analogues, specifically Tpc C). The characterisation of these peptide complexes is described in Supplementary Materials (Figure S1 and Table S1). After the disks were created, as described above, the disks were transferred to 4-mL glass vials for heat treatment. Five temperatures (100 °C, 125 °C, 150 °C, 175 °C, 200 °C) and two time points (1 min and 10 min) of exposure were selected. Following heat treatment, the disks were transferred to polystyrene plates in triplicate, including unheated peptide-treated disks and heated untreated disks. The plates were sterilized in chloroform before the use in antimicrobial assays. The same sample preparation was followed for the effect of ‘wet’ heat on Trc mix-treated cellulose disks. The only exception is that heat was applied as heated water at five temperatures (25 °C, 40 °C, 60 °C, 80 °C, 100 °C) exposed for 1 min. After exposure, the disks were transferred to polystyrene plates and dried at 40 °C.

### 3.7. Solvent Exposure of Trc-Cellulose

The effect of change in solvent on the adsorption of Trc mix to cellulose was determined in a similar fashion as to how it is created. A solution of Trc mix (100 µL of 50.0 µg/mL) containing the change in solvent (discussed later) was incubated with cellulose for 2 h. For desorption, pre-treated cellulose disks with Trc mix were treated with the different solvents for 2 h to allow for passive desorption. Following the incubation, the solutions were removed, and the samples washed three times with 100 µL sterile analytical quality water and dried in a 40 °C oven for 2 h. Solvents tested included a pH range (1–13), CaCl_2_ (2.5 mM and 7.5 mM), 1% *m*/*v* sodium dodecyl sulphate (SDS), 5% *m*/*v* acetic acid, 5 mM NaCl, 3% *m*/*v* citric acid, and a range of acetonitrile concentrations in analytical quality water (5–100%, *v*/*v*). Water washes were done by exposing Trc mix pre-treated cellulose disks with 100 µL sterile-filtered analytical quality water.

## 4. Conclusions

Association and activity of Trc mix were tested on a selection of laboratory and commercial materials. All the materials showed at least 80% inhibition of *L. monocytogenes*, with cellulose and six of the commercial materials (plastic tray, tissue paper, ripple carton, white plastic, green plastic, and cling film) resulting in full inhibition. The comparison between activity and amount of associated peptide showed that it does not always equate to activity. Interestingly, the best performing materials in terms of activity could be grouped based on the amount of peptide associated (5–8 µg/cm^2^). Furthermore, this group of materials consists of both hydrophilic and hydrophobic materials, namely cellulose, plastic polymers, and polylactate. This points to the peptide possibly changing conformation to better suit initial association to the different surfaces, which could seed further association via oligomerisation.

Tests toward the robustness of activity of Trc mix-treated cellulose showed that the material is very heat-stable, only showing a decrease in activity at 10-min exposure at 200 °C. The peptide also remains associated and active after different solvent challenges which not only shows its stability but also aids in determining the parameters for the possible commercial creation of Trc-containing materials. The only two conditions that resulted in a decrease in activity were 1% *m*/*v* SDS and 70% *v*/*v* acetonitrile, highlighting that the amphipathic nature of the Trcs and, therefore, also oligomerisation [57,58,59,71,74,77] play a role in the association with cellulose, but the fact that some activity remained could be due to the specific interaction with cellulose [53]. Finally, the uniqueness of the Trcs association to cellulose was determined by comparing the activity with other peptides at the same conditions. It was found that Trcs (Tcn, Trc mix, and Trc extract) outperformed the other peptides at the low concentrations needed to result in full inhibition of *L. monocytogenes*. GS was the only compound tested that could fully inhibit both the viability of *L. monocytogenes* and *E. coli*. It can, therefore, be used in conjunction with Trcs to create a broad-spectrum active material. 

Moreover, the assay used to assess the activity of these materials makes use of a ten-fold higher cell count compared to other solid surface methods [94,95]. This higher cell count allows for a shorter time frame for results but also allows for the selection of only the most potent surface combinations. Therefore, the full inhibition at 10^6^ cells/cm^2^ for Trcs-treated cellulose far outperformed what is expected of a material to be considered active at 10^5^ cells/cm^2^ [95].

## 5. Patents

Rautenbach M., van Rensburg, W. Method for preventing or treating microbial growth on a manufactured product. (PCT patent application published as WO2015186058A1). National validations in South Africa (ZA2016/08601, Granted); China (CN106793774, Granted); Australia (AU2015270120—granted); USA (US15,315,755, Granted); European Patent Office (EPO) (EP3151667—in process) claiming priority from ZA 2014/04023 filed 3 June 2014.

## Figures and Tables

**Table 1:** Summary of the materials used in this study as base materials.

Material Name	Application	Chemical Composition	General Chemical Character
GSWP	Solvent filtration	Mixed cellulose esters	Hydrophilic, porous
HAWP			Hydrophilic, porous
PC	General sterile filtration	Polycarbonate	Hydrophobic, porous
CN		Cellulose nitrate	Hydrophilic, porous
CL		Cellulose	Hydrophilic, porous
BP	Fluorescence assays	Polystyrene	Hard plastic
AP	Micro-dilution assays	Polystyrene	Hard plastic
PPR	Micro-dilution preparation plate	Polypropylene	Hard plastic
PSR		Polystyrene	Hard plastic
Plastic tray	Fruit packaging	Unknown	Hydrophobic, Hard plastic
Tissue paper	Fruit wrapping	Cellulose	Hydrophilic, layered cellulose
Ripple carton	Fruit packaging	Cellulose	Hydrophilic, layered cellulose
Carton box	Fruit shipping/transport	Cellulose	Hydrophilic, multiple layered cellulose
Meat paper- PL	Meat wrapping	Cellulose & polylactate	Hydrophobic
Meat paper	Meat wrapping	Cellulose	Hydrophilic
White bag	Waste bag—home use	Polylactate	Hydrophobic
Green bag	Waste bag—industrial use	Polylactate	Hydrophobic
Cling film	Food wrapping	Polylactate	Hydrophobic

**Table 2:** Summary of the compounds used to compare with the Trc in creating robust non-covalent antimicrobial materials.

Compound	Origin	Character	Minimum Inhibitory Concentration (μg/mL)	
			*L. monocytogenes*	*E. coli*
Tcn	Peptide complex produced by *Brevibacillus parabrevis*	Mixture of cationic cyclo-decapeptides and neutral pentadecapeptides, soluble in ≥50% *v*/*v* acetonitrile/organic solvents	21 ± 0.10 [34]	Not available
Trc mix	Purified from Tcn	Cationic, cyclic decapeptide complex, soluble in ≥50% *v*/*v* acetonitrile/organic solvents	23 ± 0.63 [34]	>100 [34]
GD	Purified from Tcn	Neutral linear pentadecapeptide complex, haemolytic, soluble in organic solvent	19 [83]	9 [83]
GS	*Aneurinibacillus migulanus*	Cationic, amphipathic cyclo- decapeptide, 50% identity to tyrocidine A, haemolytic, water-soluble	11 ± 0.20 [34]	3–12.5 [84]
Magainin 2	Skin of *Xenopus laevis*	Linear 23-mer cationic, amphipathic α-helical peptide, water soluble, non-haemolytic, water-soluble	Not available	10–50 [85]
PGLa	Skin of *Xenopus laevis*	Linear 21-mer cationic, amphipathic α-helical peptide amide, haemolytic, water-soluble	Not available	10–50 [86]
Leucocin A	*Leuconostoc gelidum* UAL187	37-mer cationic, amphipathic disulphide-bonded bacteriocin, non-haemolytic, water-soluble	0.98–1.98 [87]	Not available
Melittin	Venom of European honey-bee (*Apis mellifera*)	Linear 26-mer cationic, amphipathic α -helical peptide, haemolytic, water-soluble	0.315 ± 0.008 [88]	15–42.5 [89,90]
Gentamicin	*Micromonospora purpurea*	Water-soluble aminoglycoside antibiotic	0.5–4.0 [91,92]	0.156–1.25 [93]

## Data Availability

Raw data available from authors.

## Supplementary Materials

The following supporting information can be downloaded at: https://www.mdpi.com/article/10.3390/antibiotics11020174/s1, Figure S1: UPLC-MS chromatogram overlay to compare the difference in analogues present in the commercial Trc mixture, a Trp supplemented culture extract (Trp-rich peptide complex) and a Phe supplemented culture extract (Phe-rich peptide complex).; Figure S2: The fluorescence of 4 mM Phe, Tyr and Trp as detected at excitation (A) Ex_260_ (B) Ex_275_ (C) Ex_290_; Figure S3: Standard curves of Trc mix in 5% ACN at (A) Ex_290_/Ems_342_ (B) Ex_290_/Ems_350_ (C) Ex_275_/Em_324_ (D) Ex_275_/Em_342_; Figure S4: Standard curve (at Ex_290_/Ems_342_) of Trc mix in 5% acetonitrile used to calculate the amount of peptide remaining in the incubation solution by using linear regression to fit the curve (R^2^ > 0.99); Table S1: Percentage contribution of each of the tyrocidines and tyrocidine analogues as found in the commercial Trc mix, non-supplemented (Trc complex), Trp- and Phe-supplemented culture extracts (Trp-rich and Phe rich peptide complexes); Table S2: Summary of statistical analysis between Trc mix and lab acquired materials for the activity against *L. monocytogenes*; Table S3: Summary of statistical analysis between Trc mix and commercial materials for the activity against *L. monocytogenes*; Table S4: Summary of the statistical analysis between the activities observed for antibiotic treated cellulose against *L. monocytogenes*; Table S5: Summary of the statistical analysis between the activities observed for antibiotic treated cellulose against *E. coli* [96,97,98,99,100].
