# Supplementary material for: Creating Robust Antimicrobial Materials with Sticky Tyrocidines
Source: Antibiotics (Basel). 2022 Jan 28;11(2):174. doi: 10.3390/antibiotics11020174 (PMC8868332; doi:10.3390/antibiotics11020174)
Supplement: Supplementary file 1 [file antibiotics-11-00174-s001.zip › antibiotics-1561508-supplementary.pdf]

antibiotics

MDPI

Article

Creating Robust Antimicrobial Materials with Sticky Tyrocidines

Wilma van Rensburg * and Marina Rautenbach *

BIOPEP™ Peptide Group, Department Biochemistry, Stellenbosch University, Private Bag X1, Matieland, Stellenbosch 7602, South Africa

* Correspondence: wvr@sun.ac.za (W.v.R.); mra@sun.ac.za (M.R.); Tel.: +27-21-808-5872/8 (W.v.R.)

Purification of tyrocidines and analogues from commercially obtained tyrothricin and amino acid supplemented cultures

Commercial tyrocidines (Trc mix) was purified from commercially available tyrothricin as described previously [34]. An optimized method for the large-scale production of tyrocidines and supplemented cultures was employed to obtain the Phe- and Trp- containing tyrocidine analogues [96]. Characterization and purity determination of the peptide extracts were performed with a UPLC-MS method previously described [37, 96].

The linear gramicidins, produced with tyrocidines as the peptide complex tyrothricin, were removed from commercially obtained peptide with the use of an organic solvent extraction to obtain a tyrocidine extract, which is referred to as Trc mix. The main peaks detected, were Trc C1 (1361.6785), Trc C (1347.6694), Trc B1/B1′ (1322.6669), Trc B (1308.6517), Trc A1 (1283.6587) and Trc A (1269.6412). Smaller peaks of Trp C (1370.6827), Trc B′ (1308.6517), Tpc B1 (1345.6759), Tpc B (1331.6633) and deaminated Trc A (1270.6364) was also detected (Fig. S1, Table S1). No other peaks, other than solvent and machine background was detected thus the peak areas of each peptide and peptide analogues was used to determine the percentage contribution to the overall sample and subsequently the sample purity. The peptide purity was found to be >90%, thus the sample could be used as an assay control and for biophysical experiments.

The results obtained for the commercially purified peptides overall compared well to literature in terms of retention times and peptide profile [38, 97] Amino acid supplemented cultures were used to produce two different mixtures of tyrocidine analogues. The tryptophan supplementation would yield a Trc C analogue rich extract, specifically Tpc C, contrary to the Phe supplemented culture extract since it normally contains more Trc A. Both are different to Trc mix which contains a mixture of the main Trcs: Trc A/A1, Trc B/B1 and Trc C/C1. The direct comparison of the chromatogram for each of these extracts (Fig. S1) shows the shifts in the type of analogue produced. The relative purity of the fractions and the percentage contribution of each of the tyrocidine analogues were calculated by determining the peak area of each of the peptides (Table S1).

The bulk of the peptides present in the Trp-supplemented extract (Table S1) are Tpc C (43.5% contribution), Tpc C1 (26.5%) and Tpc B (10.7%). A small amount of Trc C/C1 (11.8%) was produced, with very little Trc B/B1 (±2%) and a negligible amount of Trc A and Trc A analogues. The main peptides detected in the Phe-supplemented extract were Trc A (30.2%), Trc A1 (17.3%), Trc B (15.2%) and Phc A (11%). The latter was not detected in either Trc mix or the Trp-supplemented culture extract. There remaining 30% of the extract comprised of a mixture of tyrocidines and tryptocidines. The main peptides found in the non-supplemented extract were predominantly Trc B (22.9%), Trc B1 (13.2%) and Tpc B/B′ (12.3%). Followed by Trc A (9.8%), Trc A1 (9.2%) and Trc C (8.9%).

The difference in profile, however, between the supplemented extract and Trc mix is due to the variability of the tyrocidine structure at positions 3,4 and 7. The polyenzyme unit that is responsible for the production of these peptides have low selectivity when incorporating structurally similar amino

acids at these positions [98-100]. Thus, supplementation of Trp into the growth media leads to an increased incorporation of Trp into the dipeptide unit at position 3 and 4 leading to an increase of production for Trc B- and Trc C-analogues. It also increases the incorporation of Trp at position 7 resulting the in the increased production of the tryptocidines (Tpcs). The opposite is true for Phe supplementation resulting in the increased production of Trc A analogues. The profile shifts that were obtained for the supplemented cultures aligned with the amino acid-induced shifts reported in literature [96, 98-100].

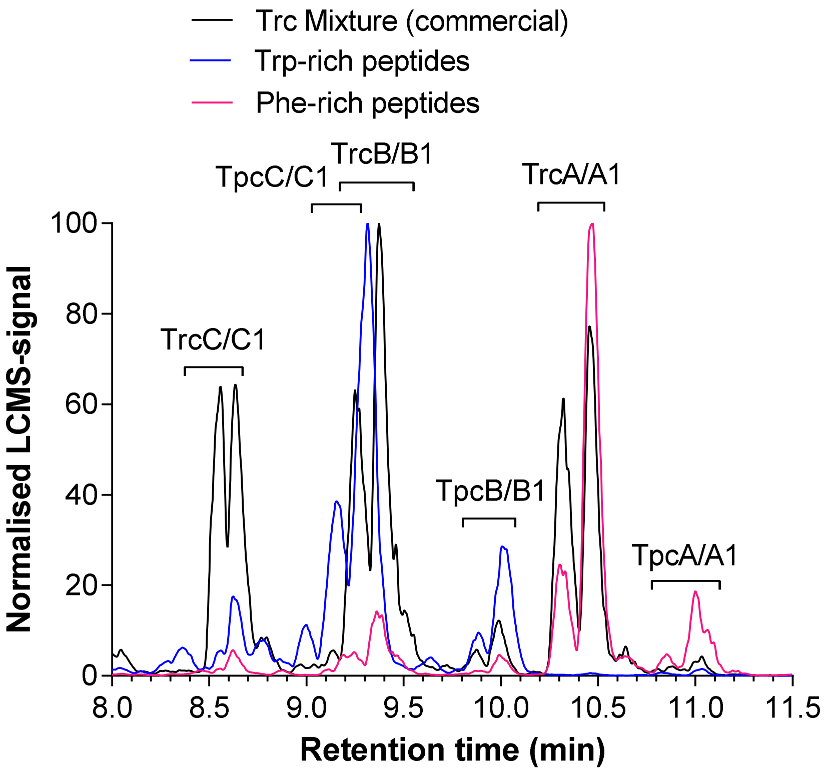

Figure S1. UPLC-MS chromatogram overlay to compare the difference in analogues present in the commercial Trc mixture, a Trp supplemented culture extract (Trp-rich peptide complex) and a Phe supplemented culture extract (Phe-rich peptide complex). The major tyrocidines groups are indicated to show the profile shift that occurs with amino acid supplementation.

Table S1. Percentage contribution of each of the tyrocidines and tyrocidine analogues as found in the commercial Trc mix, non-supplemented (Trc complex), Trp- and Phe-supplemented culture extracts (Trp-rich and Phe rich peptide complexes). The predominant tyrocidines are indicated in bold.

Peptide Identity	Sequence	M_r [c] (Theoretical)	% Contribution			
			Trc mix	Trc complex	Trp rich	Phe rich
Trc C1	*cyclo*-(fPWwNQYVKL)	1361.8404 (1361.6921)	12.0	4.6	3.0	1.5
Trc C	*cyclo*-(fPWwNQYVOL)	1347.9304 (1347.6764)	**13.9**	8.9	8.8	3.4
Tpc C1	*cyclo*-(fPWwNQWVKL)	1384.6404 (1384.7080)	1.6	2.8	**26.5**	1.2
Tpc C	*cyclo*-(fPWwNQWVOL)	1370.6844 (1370.6924)	1.9	5.1	**43.5**	2.9
Trc B1/B1′	*cyclo*-(fPFwNQYVKL)	1322.5864 (1322.6812)	**19.7**	**13.2**	<1	5.9
Trc B/B′	*cyclo*-(fPWfNQYVOL)	1308.6164 (1308.6555)	**18.4**	**22.9**	1.6	**15.2**
Tpc B1/B1′	*cyclo*-(fPWfNQWVKL)	1346.6901 (1345.6971)	1.1	3.6	5.5	<1
Tpc B/B′	*cyclo*-(fPFwNQYVOL)	1331.5744 (1331.6815)	3.4	**12.3**	10.7	4.2
Trc A1	*cyclo*-(fPFfNQYVKL)	1283.6464 (1283.6703)	**14.6**	**9.2**	<1	**17.3**
Trc A	*cyclo*-(fPFfNQYVOL)	1269.6684 (1269.6538)	12.3	9.8	<1	**30.2**
Tpc A	*cyclo*-(fPFfNQWVOL)	1292.6644 (1292.6479)	1.1	7.7	<1	6.3
Phc A	*cyclo*-(fPFfNQFVOL)	1253.6782 (1253.6597)	<1	<1	<1	**11.0**

[a] Trc - tyrocidine, Tpc – tryptocidine, Phc – phenycidine.

[b] [a]Peptide sequences, as previously described (Tang, Thibault and Boyd, 1992) where the amino acids are represented by conventional one-letter abbreviations except for Ornithine which is represented by O. The L-amino acids are in uppercase and the D-amino acids in lowercase

[c] The identity of the peptides were confirmed with high resolution mass spectrometry, with experimental Mr (top value) a representative analysis determined on the commercial Trc mixture

[d] Percentage contribution was calculated by determining the peak area as determined with LCMS for each analogue. The percentage contribution to peak area was calculated assuming equal response factors for each of the analogues.

Optimization of fluorescence parameters

Previous studies on the binding of Tyrocidines and tyrocidine analogues to various solid surfaces used changes in UV before and after incubation as the main method of determining peptide binding at 230, 256 and 280nm selecting for peptide bonds, aromatic rings and tryptophan [61]. The major drawback with this method was the occurrence of an increase in UV-signal after incubation of the peptide and some of the solid surfaces due to a release of compound(s) from the solid surface. As a result, the amount of peptide bound to the solid surfaces could not be calculated. This was especially true for cellulose-containing solid surfaces and more so for cellulose/paper. Thus, the method of detection moved from UV to fluorescence, making use of the naturally occurring fluorophores within the peptides namely Trp and Phe; and Tyr at positions 3&4 and 7 respectively. The first step in optimising this method was to determine where the fluorescence peaks would occur on the emission spectra and if any of the peaks of the three amino acids overlapped. Equimolar amounts of Trp, Tyr Phe were tested to mimic the average occurrence of these amino acids within the tyrocidine structure in terms of the three main analogues: Trc A ($F^3F^4Y^7$), Trc B ($F^3W^4Y^7$ or $W^3F^4Y^7$) and Trc C ($W^3W^4Y^7$).

All three amino acids were excited at Ex_{260}, Ex_{275} and Ex_{295}, for Phe, Tyr/Trp and Trp respectively (Fig. S2). At Ex_{260}, scanning for emission between 290 nm to 360 nm (Fig. S2: A), the fluorescence detected for Phe was similar to that of the background signal and therefore negligible. Tyr fluorescence alone was detected between 355-360 nm and Trp fluorescence at 300-302 nm. Overall, the fluorescence detected was much lower in comparison to that observed at Ex_{275} and Ex_{295}. Excitation at 275 nm, scanning from 300-360 nm, though used in the detection of Tyr also excites Trp. Therefore, any excitation of a protein or peptide between 275-280 nm, would more correctly detect both Tyr and Trp residues. This can be seen with the overlapping fluorescence detected for Tyr and Trp at Ex_{275} (Fig. S2: B). The signal for Phe was once again the same as the background and negligible. At Ex_{290}, scanning emission from 320-370 nm, predominantly Trp was detected as was expected. The signal for Tyr and Phe was negligible.

Based on the observed fluorescence, four parameters were selected: Ex_{275}/Ems_{324} (high Tyr fluorescence); Ex_{275}/Ems_{342} (high Trp fluorescence); Ex_{290}/Ems_{342} (highest signal intensity) and Ex_{290}/Ems_{350} (previously used in single fluorescence experiments) [68]. It was observed that excitation at 290nm (Fig. S3: A&B) gave better linearity, especially at low peptide concentrations in comparison to excitation at 275nm (Fig. S3: C&D). Also, the fluorescence at Ex_{290}/Ems_{342} (Fig. S3: A) gave a better fit over the entire curve compared to that observed for Ex_{290}/Ems_{350} (Fig. S3: B).

To study the behaviour of the Trcs in different environments Ex_{280} and $Ems_{300-400}$ were selected since they would detect both Trp and Tyr residue fluorescence. As for following the association and dissociation of the peptide Ex_{290} and Ems_{342} was selected as it was the most sensitive and could detect smaller changes in signal. This limits detection to only Trc C and Trc B analogues, but the assumption for this study was that all the analogues would associate and dissociate to the cellulose similarly. This assumption is based on that the organism produces the mixture of peptides close to sporulation to bind to the spore capsule (saccharide based) and therefore would bind similarly to cellulose. Calculations on the amount of peptide bound to the various surfaces were done by making use of a standard curve of Trc mix in 5% acetonitrile (Fig. S4), under the same conditions as the peptide incubation with the materials.

At one hour of incubation, the limit of detection was at 5 μg/mL (S/N>2, 0.5 μg Trc mix) and the limit of quantification was at 10 μg/mL (S/N>10, 1 μg Trc mix). The limit of linearity was at 50 μg/mL, which was the highest concentration tested in the standard curve, but also the concentration used for the treatment of the solid surfaces. A fit of $R^2 > 0.99$ was found and the amount of peptide bound was calculated by using the linear equation presented on the graph.

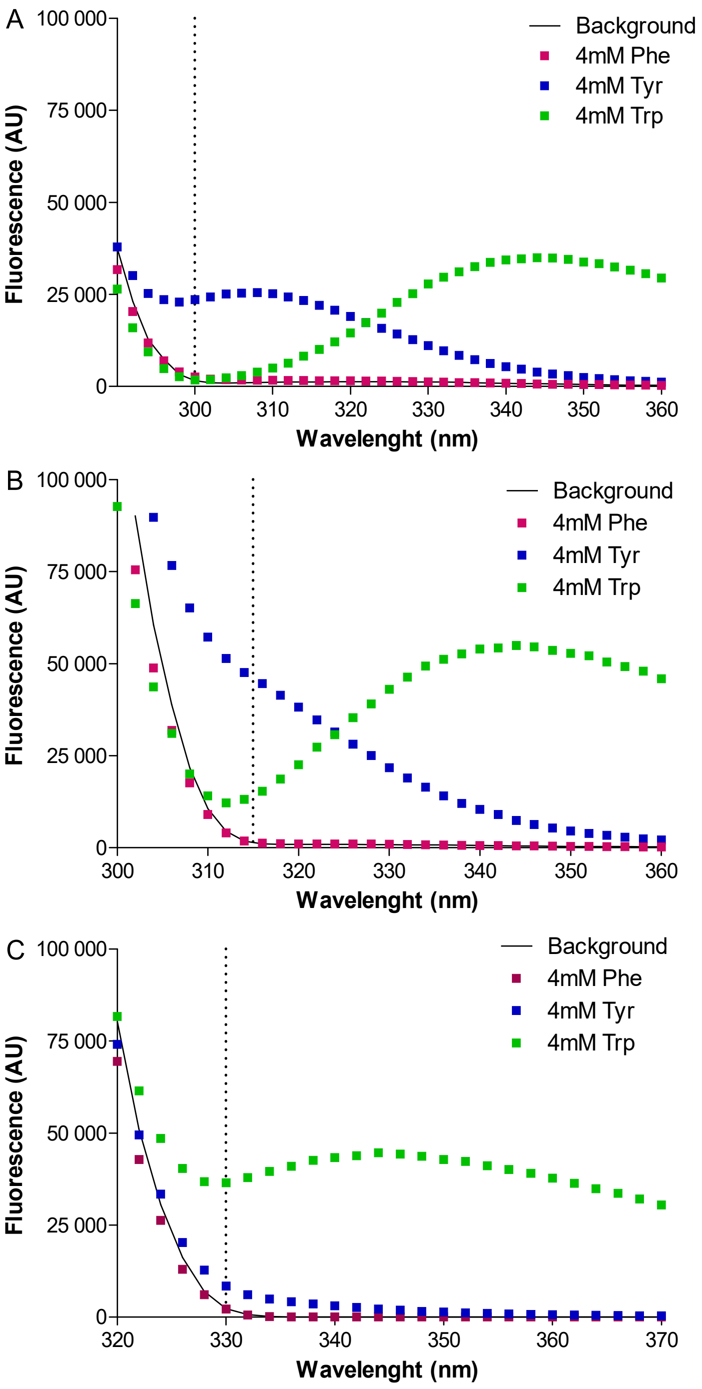

Figure S2. The fluorescence of 4mM Phe, Tyr and Trp as detected at excitation **(A)** Ex_{260} **(B)** Ex_{275} **(C)** Ex_{290}. The dotted line indicates at which point the background signal is zero. The data is representative of only one technical repeat.

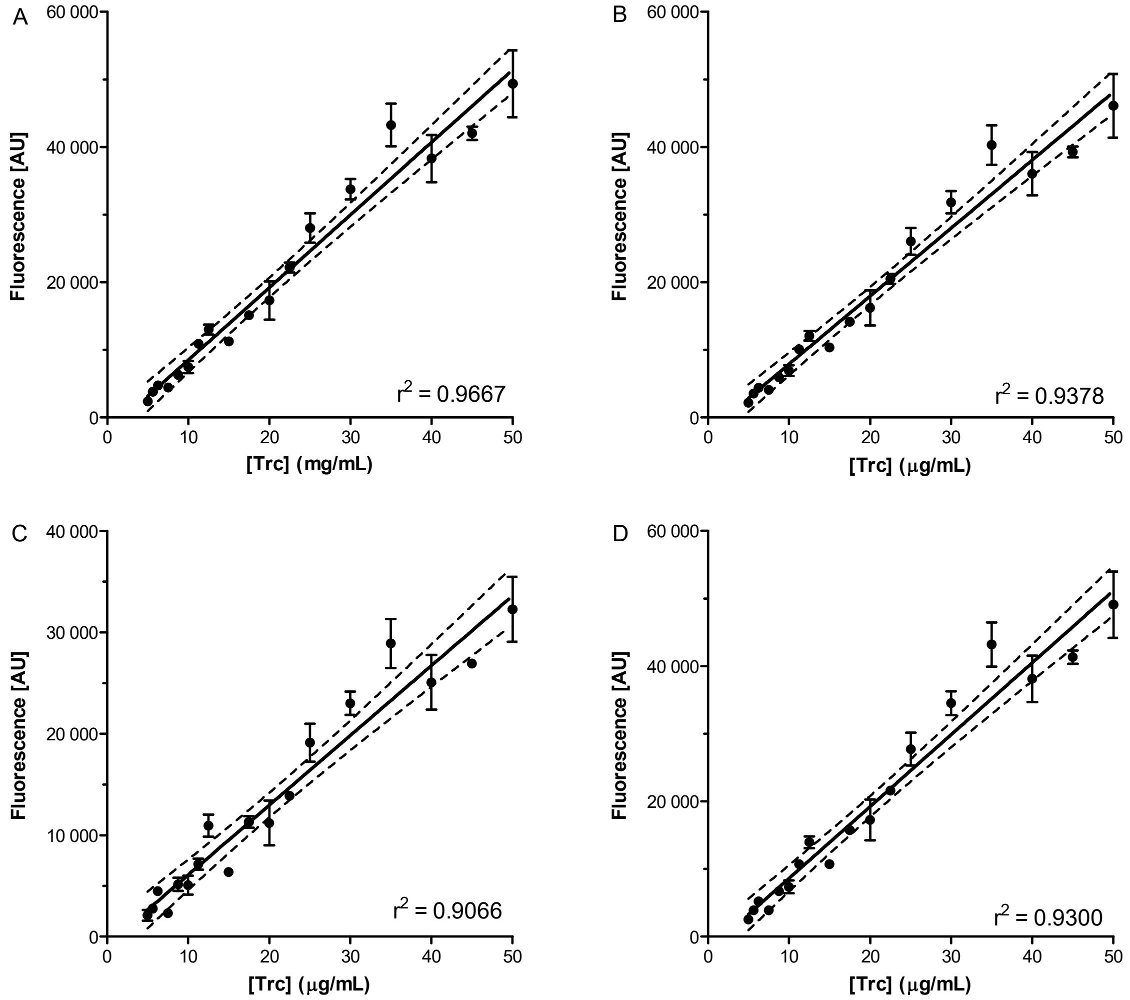

Figure S3. Standard curves of Trc mix in 5% ACN at **(A)** Ex290/Ems342 **(B)** Ex290/Ems350 **(C)** Ex275/Em324 **(D)** Ex275/Em342. Linear regression was used to fit the curves (R^2>0.95). Each datapoint is the average of triplicate determinations with error bars showing the standard error of the mean (SE). The dotted lines show the 95% confidence interval (CI) for the fitted curve (solid line).

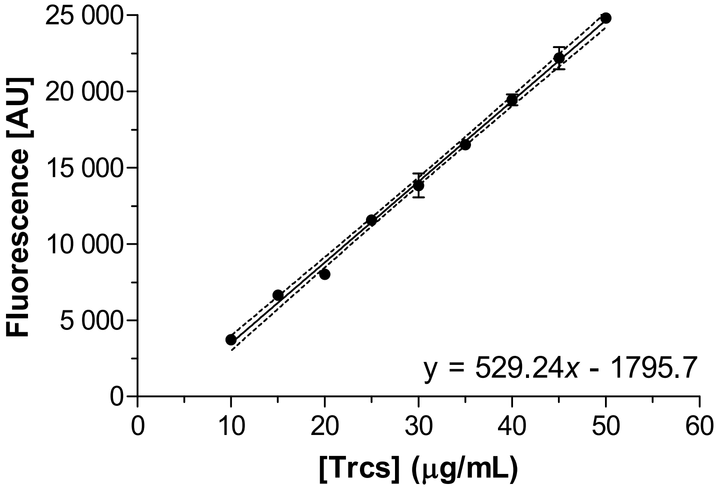

Figure S4. Standard curve (at Ex290/Ems342) of Trc mix in 5% acetonitrile used to calculate the amount of peptide remaining in the incubation solution by using linear regression to fit the curve (R^2>0.99). Each datapoint is the

average of quadruplicate determinations with error bars showing the SE. The dotted lines show the 95% CI for the fitted curve (solid line).

Summary of full statistical analysis

Table S2. Summary of statistical analysis between Trc mix and lab acquired materials for the activity against *L. monocytogenes* (Fig. 2: A). Abbreviations: cellulose (CL), mixed cellulose filters GSWP (0.22 μm) and HAWP (0.45 μm); cellulose nitrate (CN – 0.4 μm), polycarbonate (PC – 0.4 μm), black polystyrene plate (BP), polypropylene round bottom plate (PPR), polystyrene round bottom plate (PSR) and polystyrene plate (AP). Statistical analysis was performed with One-way Anova (Bonferroni's multiple comparison test). ****p* <0.001; ***p* <0.01; **p* <0.05, ns – not significant

	CL	GSWP	HAWP	CN	PC	BP	PPR	PSR	AP
Trc mix	ns	*	***	**	***	***	***	***	***
CL		**	***	***	***	***	***	***	***
GSWP			ns	ns	ns	**	**	***	***
HAWP				ns	ns	*	ns	***	***
CN					ns	*	ns	***	***
PC						ns	ns	***	***
BP							ns	***	***
PPR								***	***
PSR									ns

Table S3. Summary of statistical analysis between Trc mix and commercial materials for the activity against *L. monocytogenes* (Fig. 2: B). Statistical analysis was performed with One-way Anova (Bonferroni's multiple comparison test). ****p* <0.001; ***p* <0.01; **p* <0.05, ns – not significant

	Plastic tray	Tissue paper	Ripple carton	Paper Tray	Carton box	Meat paper – wax	Meat paper	White bag	Green bag	Cling film
Trc mix	ns	ns	ns	***	*	***	***	ns	ns	ns
Plastic tray		ns	ns	***	ns	***	***	ns	ns	ns
Tissue paper			ns	***	ns	***	***	ns	ns	ns
Ripple carton				***	ns	ns	***	ns	ns	ns
Paper tray					***	***	***	***	***	***
Carton box						ns	**	ns	ns	ns
Meat paper - wax							ns	**	**	**
Meat paper								***	***	***
White bag									ns	ns
Green bag										ns

Table S4. Summary of the statistical analysis between the activities observed for antibiotic treated cellulose against *L. monocytogenes* (Fig. 6: A). Abbreviations: GD – Linear gramicidins, GS- gramicidin S. Statistical analysis was performed with One-way Anova (Bonferroni's multiple comparison test). ***p <0.001; ns – not significant

	Trc mix	GD	Trc Extract	GS	Magainin 2A	PGLa	Leucocin A	Melittin	Gentamycin
Tyrothricin	ns	***	ns	ns	***	***	***	***	***
Trc mix		***	ns	ns	***	***	***	***	***
GD			***	***	***	ns	***	***	***
Trc Extract				ns	***	***	***	***	***
GS					***	***	***	***	***
Magainin 2A						***	***	ns	***
PGLa							***	***	***
Leucocin A								***	ns
Melittin									***

Table S5. Summary of the statistical analysis between the activities observed for antibiotic treated cellulose against *E. coli* (Fig. 6: B). Abbreviations: GD – Linear gramicidins, GS- gramicidin S. Statistical analysis was performed with One-way Anova (Bonferroni's multiple comparison test). ***p<0.001; ns – not significant

	Trc mix	GD	Trc Extract	GS	Magainin 2A	PGLa	Leucocin A	Melittin	Gentamycin
Tyrothricin	ns	ns	ns	***	***	***	***	***	***
Trc mix		**	ns	***	***	***	***	***	***
GD			*	***	***	***	ns	ns	***
Trc Extract				***	***	***	***	***	***
GS					***	***	***	***	***
Magainin 2A						***	***	***	***
PGLa							***	***	***
Leucocin A								ns	***
Melittin									***
